# Molecular and Culture-Based Surveillance of Free-Living Amoebae in Human Related Sources in an Outermost Region

**DOI:** 10.3390/pathogens15010073

**Published:** 2026-01-09

**Authors:** Marco D. Peña-Prunell, María Reyes-Batlle, Patricia Pérez-Pérez, Rubén L. Rodríguez-Expósito, Ines Sifaoui, Omar García-Pérez, Angélica T. Domínguez-de Barros, Elizabeth Córdoba-Lanús, José E. Piñero, Jacob Lorenzo-Morales

**Affiliations:** 1 Instituto Universitario de Enfermedades Tropicales y Salud Pública de Canarias, Universidad de La Laguna, Av. Astrofísico Francisco Sanchez, s/n, 38200 San Cristóbal de La Laguna, Spain; alu0100908206@ull.edu.es (M.D.P.-P.); pperezpe@ull.edu.es (P.P.-P.); rrodrige@ull.edu.es (R.L.R.-E.); isifaoui@ull.edu.es (I.S.); ogarciap@ull.edu.es (O.G.-P.); acordoba@ull.edu.es (E.C.-L.); 2 Departamento de Obstetricia y Ginecología, Pediatría, Medicina Preventiva y Salud Pública, Toxicología, Medicina Legal y Forense y Parasitología, Universidad de La Laguna, C/Sta. María Soledad s/n, 38200 San Cristóbal de La Laguna, Spain; 3 CIBER de Enfermedades Infecciosas (CIBERINFEC), Instituto de Salud Carlos III, 28029 Madrid, Spain; 4 Departamento de Bioquímica, Microbiología, Biología Celular y Genética, Universidad de La Laguna, 38200 San Cristóbal de La Laguna, Spain

**Keywords:** agricultural soil, playground soil, public refrigerated fountains, free living amoeba, tolerance assays

## Abstract

In this study, we investigated the presence and diversity of FLA in 62 environmental samples collected across Tenerife, Canary Islands, Spain including agricultural and playground soils, and on double treated water from public refrigerated fountains. Amoebae were isolated by culturing processed samples onto 2% Non-Nutrient Agar plates (NNA) which were checked daily for further processing up to molecular characterization. In this case, two approaches for molecular identification were assessed: direct multiplex qPCR targeting four potentially pathogenic FLA (*Acanthamoeba* spp., *Vermamoeba vermiformis*, *Naegleria fowleri*, and *Balamuthia mandrillaris*) DNA, and culture-based isolation followed by standard PCR and sequence analysis. Regarding qPCR results, 72.6% (45/62) of the samples were positive for at least one FLA, with *V. vermiformis* (37/62) and *Acanthamoeba* spp. (34/62) being the most frequent. Moreover, *B. mandrillaris* was detected for the first time in the Canary Islands in 6 out of 62 samples. Results from standard PCR from cultured isolates confirmed the presence of *Acanthamoeba* (mainly genotype T4) and *Vermamoeba* and also allowed the identification of Vahlkampfia and *Vannella* genera, as well as the genus *Rhogostoma*—its first report in the Canary Islands. Thermotolerance and osmotolerance assays were performed on *Acanthamoeba* spp. and, innovatively, on *V. vermiformis* isolates. Both were capable of surviving at 37 °C and during incubation with 0.5 M mannitol, suggesting potential pathogenicity. However, growth was significantly impaired under harsher conditions (42 °C and 1 M mannitol). These findings underscore the widespread occurrence of FLA in public and agricultural environments in Tenerife and highlight their potential risk to public health. Their ability to act as carriers of pathogenic bacteria/viruses further reinforces the need for routine surveillance and preventive measures in the environment.

## 1. Introduction

Free-living amoeba (FLA) are widely distributed protozoa found in water, soil, or dust. These organisms are amphizoic and live on organic matter or other microorganisms. However, some of these FLA are able to act as opportunistic parasitic pathogens in humans and other animals [1]. Most FLA species, depending on external conditions, manifest two stages in their life cycle. Under favorable conditions, they assume the trophozoite form, acquiring mobility through pseudopodia, which they use for feeding and reproduction. When conditions become unfavorable, they turn into a cyst form, surrounding themselves with a protective wall that grants them resistance against environmental hazards [2].

Furthermore, FLA play an important role in microbial ecology. By acting as predators, they help to regulate microbial populations, especially bacterial ones, keeping a healthy rate of organic matter mineralization in the environment [3]. FLA’s role in bacterial consumption, floc formation, and ecological regulation suggests that amoebas are potentially important contributors to the efficiency and stability of aerobic sewage treatment systems [4].

FLA belonging to the genus *Acanthamoeba*, or the species *Vermamoeba vermiformis*, *Balamuthia mandrillaris*, and *Naegleria fowleri* have been described as emerging pathogenic amoebae [1,5]. In addition, it is important to mention that *Acanthamoeba* is the most common FLA found in environmental samples [6]. Based on the nucleotide sequence of the Diagnosis Fragment 3 (DF3) from the ribosomal RNA18S internal region, this genus currently consists of 23 genotypes (T1–T23). Among them, genotype 4 is the most common in both environmental and clinical samples [7]. This genus can cause keratitis if they colonize the corneal epithelium [8] or granulomatous amoebic encephalitis if they invade the central nervous system mostly in immunocompromised individuals [9].

Likewise, *V. vermiformis* is considered a thermotolerant amoeba which also presents a high prevalence in environmental samples, mainly water related sources [9]. Moreover, clinically it has been reported as the causative agent of encephalitis, skin epithelial disorders, and keratitis, alone or in coinfection with *Acanthamoeba* or *Vahlkampfia* genera [8,10,11,12].

In contrast, the thermophilic FLA *Naegleria fowleri* has been predominantly documented on warm freshwater bodies [13]. This pathogenic amoeba causes primary amoebic meningoencephalitis (PAM), a rare but highly fatal disease [14]. By invading the central nervous system, it can affect even immunocompetent individuals. It is capable of multiplying at temperatures up to 45 °C and, in addition to the usual trophozoite and cyst forms, presents a third transitional flagellate stage with increased mobility [1,15].

*Balamuthia mandrillaris* causes ulcer-like lesions or swellings in contact with the skin that do not resolve with conventional treatments [1]. Over time, these skin lesions can provide an entry point for the amoeba to enter the bloodstream and eventually the central nervous system, leading to a highly fatal form of granulomatous amoebic encephalitis (GAE). If the initial invasion occurs through the nasal route or pre-existing open wounds, the time between infection and death is drastically reduced, sometimes even less than a week [16]. Nevertheless, the exact routes of invasion of this pathogen are still not fully understood and further studies are needed.

Risk groups implicated in FLA infections include people with weakened immune systems, children and adolescents, especially those involved in recreational water activities, as well as people who work directly with soil, such as farmers and gardeners. If adequate prevention actions are not taken, common scratches or cuts on the skin would provide an entry point for these microorganisms [17].

In addition to their pathogenic capacity, they also pose an indirect risk when ingested, as some foodborne pathogenic bacteria can survive amoeba phagocytosis, using FLA as protective carriers, especially when FLAs adopt the cyst form [18]. Notable examples include *Salmonella enterica* and *Vibrio cholerae*, which can survive control measures such as antibiotics or biocides when internalized within FLAs, facilitating their entry and infection of humans [19]. This is particularly relevant for foods that are susceptible to FLA contamination and are commonly consumed with minimal processing, such as vegetables and leafy greens that come into direct contact with nutrient-rich agricultural soils or irrigation water [18].

Considering all these aspects, the purpose of our work was to appraise and characterize FLA in human-related environments. Tenerife, the largest of Spain’s Canary Islands archipelago, is located off the northwest coast of Africa and characterized by a subtropical climate and diverse ecosystems. These environmental conditions, combined with extensive human activity, create favorable habitats for FLA. In order to assess this objective, we focused on the current study in agricultural and playground soils, and on double treated water from public refrigerated fountains.

## 2. Materials and Methods

### 2.1. Geographical Location

**Figure 1:**
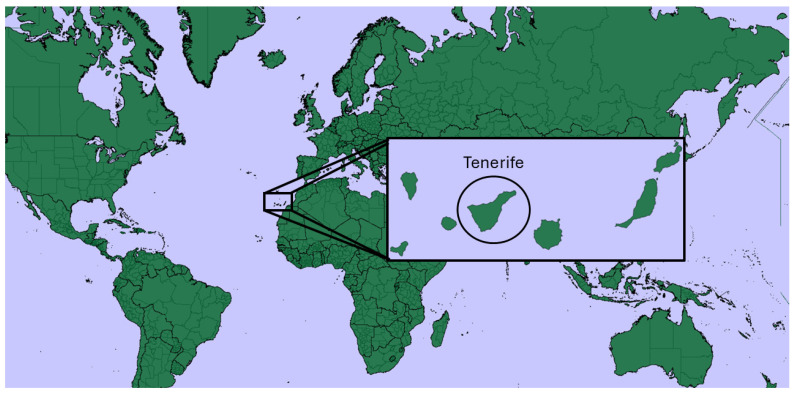
Canary Islands and Tenerife Location. Figure generated on Microsoft PowerPoint using rights free maps.

A total of 62 (45 soil and 17 water) samples were collected across the island of Tenerife (Canary Islands, Spain, 28°16′7″ N 16°36′20″ W [Figure 1]). Regarding soil samples, 25 belonged to active agricultural soils (AS) and 20 were recovered from children’s playgrounds (PS). Related to water samples, 17 refrigerated drinking water fountains (RW) were monitored. Samples from playgrounds and refrigerated drinking water were collected from various locations within the metropolitan area of Tenerife (the municipalities of Santa Cruz de Tenerife and San Cristóbal de La Laguna). However, due to the scarcity of agricultural soil in urban areas, we covered a total of 18 different municipalities around the island.

### 2.2. Sample Collection and Processing

**Figure 2:**
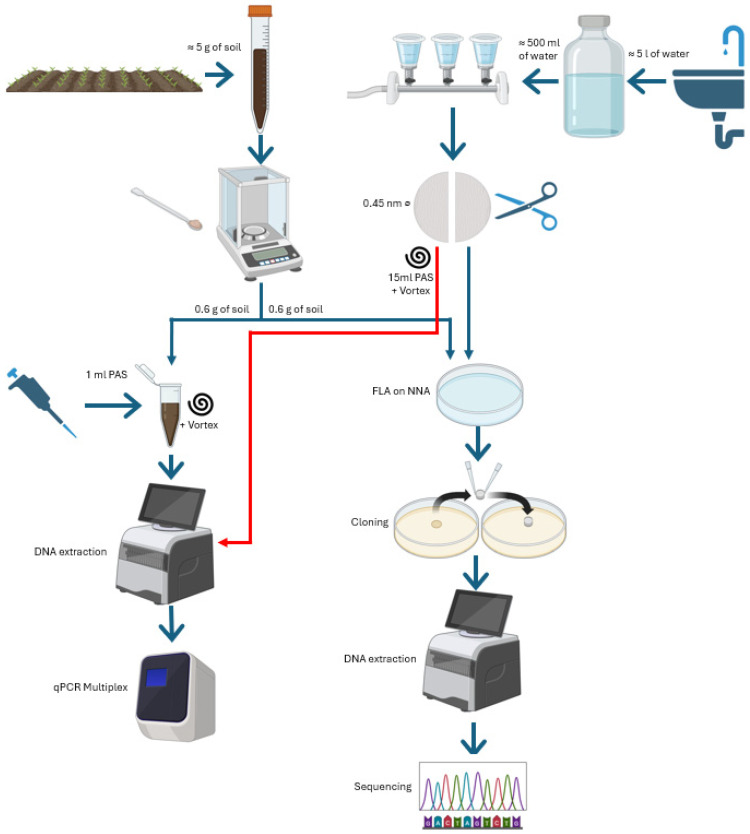
Workflow scheme for FLA molecular characterization from environmental samples. Figure created in BioRender. Peña Prunell, M. (2025) https://BioRender.com (accessed on 1 September 2025).

In order to obtain homogeneous samples, soil was collected from three different and randomized points within the same sampling area. Approximately 5 g of soil per zone were taken and introduced into 15 mL sterile tubes and stored at 4 °C until processed. On the other hand, for the refrigerated drinking water samples, a total of 5 L was collected per drinking fountain and kept into sterile flasks at 4 °C until its processing. The initial sample handling, as well as the subsequent molecular analysis of them, are depicted in Figure 2.

From each soil sample, 0.6 g of soil was reserved for the most pathogenic FLA detection by a simultaneous Multiplex qPCR (detailed below). On the other hand, another 0.6 g soil fraction was plated on 2% non-nutrient agar (NNA) under sterile conditions.

Related to water sources, samples were filtered through a 0.45 μm nitrocellulose membrane, which was then divided into two halves. Following the same process that was used for soil samples, one half was resuspended in 15 mL of PAS, vortexed and removed. The suspension was centrifuged, and the pellet was used for DNA extraction and subsequent multiplex qPCR. Meanwhile, the other half was plated on 2% non-nutrient agar (NNA) and incubated under sterile conditions.

To obtain monoxenic amoeba cultures, all NNA plates were incubated at room temperature and monitored daily using the Leica DMi1 inverted microscope (Leica Camera AG, Wetzlar, Germany). Amoebae growing in the cultures were isolated through cloning by cutting out the selected section and transferring onto a fresh NNA plate [16]. For the initial inoculation of each sample, only NNA plates were used, in order to ensure that the endogenous bacteria present in each sample served as the substrate for the amoebae. For subsequent cloning steps, plates overlaid with inactivated *Escherichia coli* were used as the substrate to prevent complications associated with potentially low initial bacterial loads.

### 2.3. Amoebae Molecular Characterization

Two different techniques were used for this purpose, a multiplex qPCR for the most pathogenic FLA detection, and standard PCR for the genotyping of the isolated FLA species. Both molecular approaches used in this study have already been optimized and validated in previous works by our laboratory [20,21]. Concerning the first task, 0.6 g of soil weighed and resuspended into 1 mL of Page’s Amoeba Solution (PAS) [22]. To continue obtaining a representative sample characterization, 0.6 g of soil was weighed three times in 0.2 g portions, with the container being shaken energetically each time. This suspension was used for DNA extraction using the semi-automatic instrument Maxwell RSC (Promega, Madrid, Spain) and processed as per manufacturer’s instructions. The obtained DNA was subjected to a Multiplex qPCR to detect the presence of DNA of *Acanthamoeba* spp., *Naegleria fowleri*, *Vermamoeba vermiformis* and *Balamuthia mandrillaris* [23]. Nevertheless, in order to obtain DNA from monoxenic culture for standard PCR, the surface of the monoxenic culture plate was covered with 4 mL of PAS and scraped to collect the cultured amoebae. This suspension was then centrifuged, and the pellet was transferred to a Maxwell RSC cell DNA Purification Kit sample cartridge (Promega, Madrid, Spain) as mentioned above.

Once isolated, the amoeba growth was identified using a morphological approach, following the guidelines in the morphological keys of F.C. Page (1988) [22], with the aim of selecting the appropriate primer set and conditions for standard PCR and DNA sequencing (Table 1 and Table 2).

In both cases, the obtained DNA yield and purity were determined using NanoDrop^®^ 1000 spectrophotometer (Fisher Scientific, Madrid, Spain). The obtained samples presented DNA concentration of 1–10 ng/ul and 260/280 ratio of ~1.8.

### 2.4. qPCR Assay

Following the procedure previously described in our laboratory [23], we simultaneously detected amoebae’s DNA of the genus *Acanthamoeba* spp., *Acanthamoeba* T4 strains, and the species *V. vermiformis*, *B. mandrillaris*, and *N. fowleri*. The primers and TaqMan probes used for *Acanthamoeba*, *V. vermiformis*, and *B. mandrillaris* were as previously reported in that study (Table S1). ParoReal kit *Acanthamoeba* T4 (Ingenetix-GmbH, Vienna, Austria) was used to determine if the *Acanthamoeba* spp. found in the preceding phase match the T4 genotype, the assay targets the 18S rRNA gene of *Acanthamoeba* species of genotype T4 (*A. castellanii*, *A. lugdunensis*, *A. mauritaniensis*, *A. polyphaga*, *A. rhysodes*, *A. royreba*).

The reaction was prepared in a final volume of 10 µL containing 10X TaqMan™ multiplex master mix (Applied Biosystems, Thermo Fisher Scientific, Waltham, MA, USA), 0.5 µM of each primer, 0.25 µM of each probe, and 2 µL of DNA sample (either water or cultured FLA strains). Amplification was performed using a QuantStudio 5 real-time PCR system (Thermo Fisher Scientific, Madrid, Spain) under the following thermal conditions: initial denaturation at 95 °C for 3 min, followed by 40 cycles of 95 °C for 15 s and 60 °C for 1 min.

Positive controls consisted of DNA extracted from axenic cultures of *Acanthamoeba castellanii* Neff (ATCC^®^ 30011™), *N. fowleri* (ATCC^®^ 30808™), and environmental isolates from our laboratory of *B. mandrillaris* (NCBI KJ439569) [16] and *V. vermiformis* (NCBI MT320010) [23]. For the T4 assays, internal DNA positive controls as well as inhibition controls were included in the ParoReal kit used. The reaction was set up in a QuantStudio 3 real-time PCR thermocycler (Thermo Fisher Scientific, MA, USA) following the manufacturer instructions.

CT values over 36 were considered negative, as CT values between 34 and 35 were determined to correspond to the DNA content of a single cell. Therefore, CT values of 36 or higher indicate the detection of few DNA copies or fragmented DNA, which is consistent with non-viable samples. This qPCR showed an efficiency (E) of 100.3% and a correlation (R^2^) of 0.96.

### 2.5. FLA Sequencing and Genotyping by Standard PCR

To perform sequencing for all positive samples, and genotyping specifically for *Acanthamoeba*, all positive samples were subjected to a PCR. Based on the initial morphological approach using the monoxenic cultures, 18S rRNA specific primers were used. Depending on each primer couple, different PCR conditions were developed (Table 1 and Table 2).

PCR products were analyzed by electrophoresis on a 2% agarose gel, and positive samples were sequenced using Macrogen Spain service (Madrid, Spain). To identify our samples, we compared our samples with >98% homologous reference strains present in the NIH GenBank database.

### 2.6. Phylogenetic Analysis

To identify isolated FLA phylogenetic relations, the MAFFT Multiple Sequence Alignment Software Version 7 was used [28] to align our samples with reference sequences from the NIH GenBank database based on homology. After alignment, RAxML version 8 was used [29] to construct a phylogenetic tree using the maximum likelihood method with 1000 bootstrap replicates, representing the possible evolutionary divergence of our samples.

### 2.7. Tolerance Assays on Potentially Pathogenic Amoeba

Osmotolerance and thermotolerance assays were performed on monoxenic cultures containing either *Acanthamoeba* spp. or *V. vermiformis*. The amoebae on NNA plates were subjected to four different tests in triplicate with approximately 10^3^ cells inoculated onto a fresh NNA plate for each assay, under the following conditions: 37 °C and 42 °C to evaluate thermotolerance, and 0.5 m and 1 m mannitol agar at room temperature, to evaluate osmotolerance [30]. After 72 h, the samples were observed and assigned a value between 0 and 3 depending on the grading system established in Table 3.

The results were analyzed statistically using R software (R 4.5.1 version) [31] to determine whether there were any significant differences between *Acanthamoeba* spp. and *V. vermiformis* strains. The experimental assays were also divided into two groups where Group 1 comprised assays conducted at 37 °C and 0.5 M mannitol (standard human conditions) and Group 2 comprised assays conducted at 42 °C and 1 M mannitol (conditions of greater stress, such as febrile states or tissues with high osmotic pressure). It was verified that the data met the assumptions of normality and homoscedasticity, and two-way and one-way ANOVAs were performed to assess differences between strains and groups, respectively.

## 3. Results

### 3.1. Pathogenic FLA Detection by qPCR

**Figure 3:**
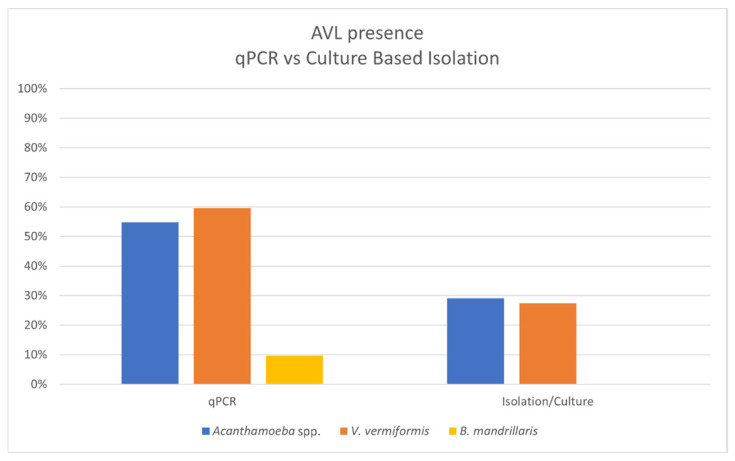
Distribution of potentially pathogenic FLA detected by qPCR vs. isolated amoebas from culture.

In the present study, multiplex qPCR assay detected at least one of the most pathogenic FLA in 45 of the 62 (73%) samples. These positive samples are distributed as follows: AS: 15/25 (60%); PS: 19/20 (95%) and RW: 11/17 (65%). The species *V. vermiformis* was the most commonly detected, present in 37/62 (60%) samples, followed by *Acanthamoeba* spp. with 34/62 (55%) and *B. mandrillaris* with 6/62 (10%) (Table 4, Figure 3). For complete details, refer to Table S2.

### 3.2. FLA Isolation by NNA Culture

Of the total of samples tested (62), we were able to obtain monoxenic cultures from 47 (75.8%) of the samples and these were positive for at least one type of FLA. The higher rate of positives was in agriculture soils (AS), with 96% (24/25), followed by playground soil samples (PS) (15/20; 75%) and RW: 7/17 (35.3%). For complete details, refer to Table S3.

**Figure 4:**
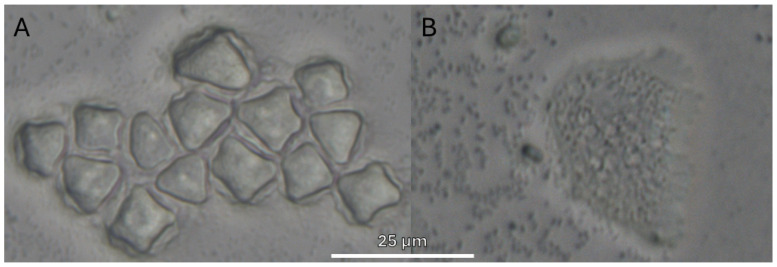
*Acanthamoeba* cells. (**A**): Several *Acanthamoeba* cysts, ranging between 15 and 20 µm in diameter. A characteristic double wall with the inner wall displaying polygonal morphology was observed. (**B**): Granular, irregularly shaped trophozoite with acanthopodia and vacuolated cytoplasm. Images (40×) were obtained with an ECHO Revolution hybrid microscope. The scale bar corresponds to 25 µm.

**Figure 5:**
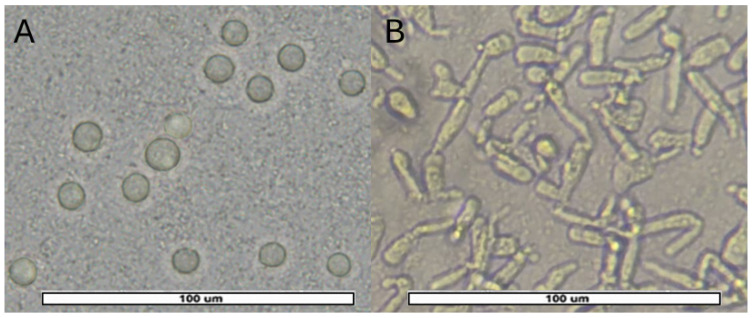
*Vermamoeba vermiformis* cells. (**A**): Spherical cysts with an approximate diameter of 5–10 µm, with a single smooth wall and a compact cytoplasm (**B**): The trophozoite shows a worm-like, elongated and slender morphology, measuring approximately 30–40 µm in length, it extends by forming a single lobose pseudopodium. Images (40×) were obtained with an ECHO Revolution hybrid microscope. The scale bars correspond to 100 µm.

**Figure 6:**
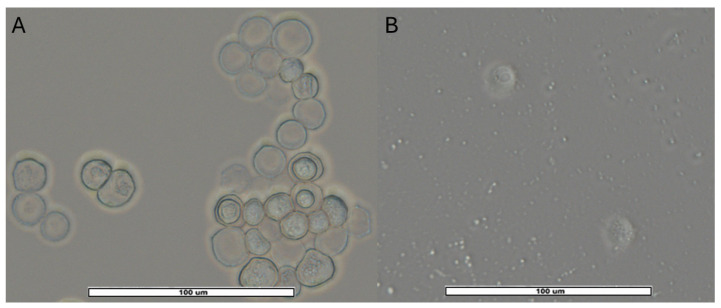
(**A**): Vahlkampfiid cysts observed in clusters, spherical to ovoid in shape, measuring approximately 15–20 µm in diameter. The outer wall is thin and smooth, and the nucleus is clearly visible. (**B**): The image shows two *Rhogostoma* trophozoites enclosed in their hyaline tests and granular cytoplasm inside. Images (40×) were obtained with an ECHO Revolution hybrid microscope. The scale bars correspond to 100 µm.

**Figure 7:**
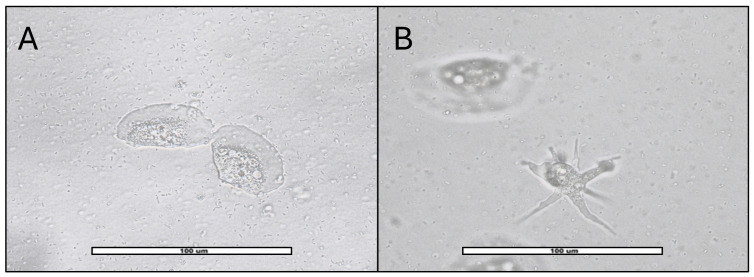
*Vannella* trophozoites from AS sample MPP25S. Images were obtained with an ECHO Revolution hybrid microscope (40×). The scale bar corresponds to 100 µm. (**A**): Two trophozoites in locomotive form, approximately 40 µm in size, surrounded by a semicircular hyaline layer resembling a fan without discrete pseudopodia. (**B**): One trophozoite in locomotive form (top side) and one trophozoite in floating form (bottom side) only observable in liquid medium, it acquires a three-dimensional structure by extending multiple pseudopods in all directions, with the granuloplasm becoming more compact. Based on our observations, it is capable of transitioning between both forms in less than 15 s after adhering to a solid surface. These morphological characteristics, along with the results obtained through PCR and sequencing, have allowed us to identify this FLA as belonging to the genus *Vannella*.

From positive samples (47), 51 monoxenic subcultures were obtained: 27 from AS, 17 from PS and 7 from RW. They were divided into a wider variety of amoebas consisting of 18/51 (35.3%) *Acanthamoeba* sp. (Figure 4); 17/51 (33.3%) *V. vermiformis* (Figure 5); 11/51 (21.6%) Fam. Vahlkampfiidae (Figure 6); 4/51 (7.8%) *Vannella* sp. (Figure 7) and 1/51 (2%) *Rhogostoma* sp. (Figure 6). These findings are consistent with global prevalence data reported in the literature [6], with *Acanthamoeba* being the most commonly detected FLA. After analysis of the DF3 fragment (rRNA 18S) of the 18 *Acanthamoeba* strains of this study, it was confirmed that 2 (11%) belonged to genotype T2, 15 (83%) to genotype T4 and 1 (5.6%) to genotype T5.

### 3.3. Phylogenetic Relationship

**Figure 8:**
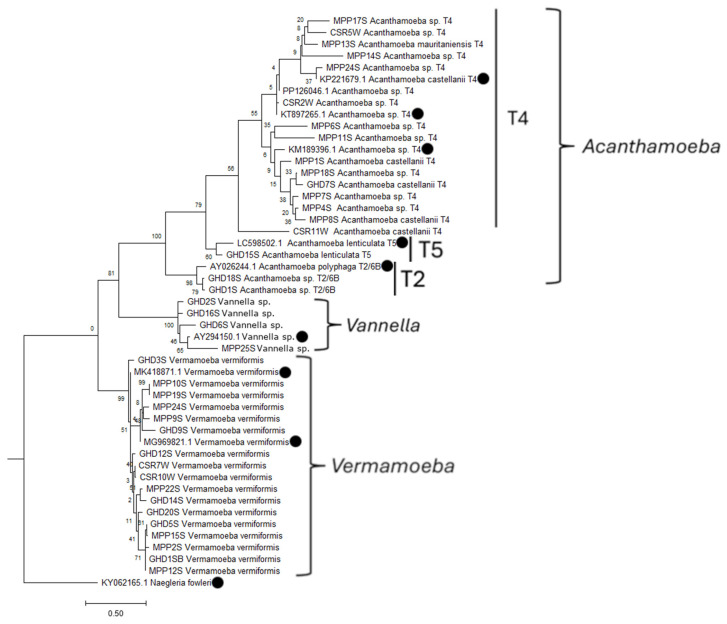
Maximum likelihood phylogenetic tree based on 18S rRNA gene sequences, inferred using RAxML with 1000 rapid bootstrap replicates. It includes 48 sequences, 10 of which are reference sequences obtained from GenBank and marked with black circles at the tips. The tree is rooted using *Naegleria fowleri* as an outgroup. Bootstrap support values are shown at the nodes, indicating the robustness of each clade. The scale bar represents 0.5 substitutions per nucleotide site, indicating the number of changes per site along the branches.

**Figure 9:**
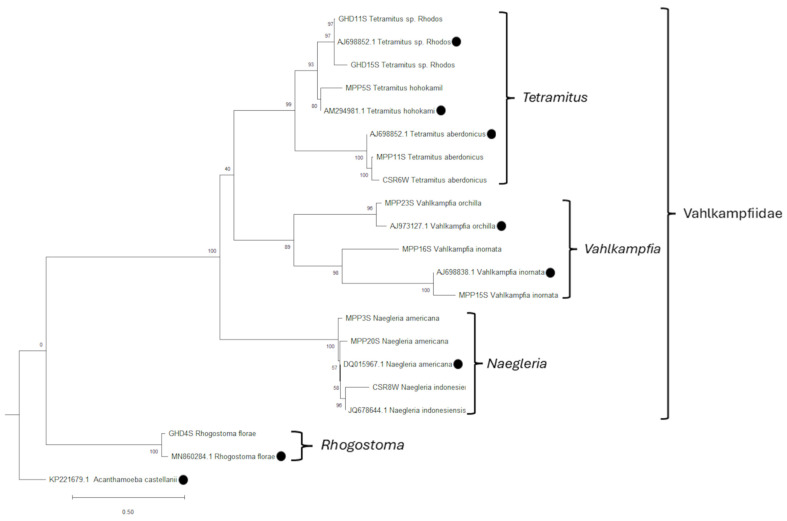
Maximum likelihood phylogenetic tree based on 5.8S gene sequences and internal transcribed sequences (ITS1 and ITS2) for the Vahlkampfiidae samples and 18S rRNA gene sequences for the *Rhogostoma* samples, inferred using RAxML with 1000 rapid bootstrap replicates. It includes 18 sequences, 6 of which are reference sequences obtained from GenBank and marked with black circles at the tips. The tree is rooted using *Acanthamoeba castellanii* as an outgroup. Bootstrap support values are shown at the nodes, indicating the robustness of each clade. The scale bar represents 0.5 substitutions per nucleotide site, indicating the number of changes per site along the branches.

Two phylogenetic trees were inferred: one for amoebae belonging to the Amoebozoa clade based on 18S rRNA gene sequences (Figure 8), and one for amoebae belonging to the Vahlkampfiidae family and the *Rhogostoma* genus based on 5.8S gene sequences and internal transcribed (ITS1 and ITS2) and 18S rRNA gene sequences, respectively (Figure 9). Regarding Figure 8, the majority of *Acanthamoeba* isolates fall within the T4 genotype, while T5 and T2 are less common and branch off separately. All *V. vermiformis* isolates belonging to Tubulinea phylum are grouped together, and are clearly apart from *Acanthamoeba* and *Vannella*, which are part of Discosea phylum [32].

On the other hand, related to Figure 9, Vahlkampfiidae species have been grouped in three main genera: *Tetramitus*, *Vahlkampfia* and *Naegleria* [33,34]. *Tetramitus* clade is well-supported and has two different species: *T. aberdonicus* and *T. hohokami*, while GH11S joins the *Tetramitus* sp. Rhodos strain. However, *Rhogostoma* sp. is a genus belonging to the monophyletic supergroup SAR [35] which only shares with the Vahlkampfiidae species that are eukaryotes, which is clearly represented in the phylogenetic tree.

### 3.4. Osmotolerance and Thermotolerance

**Figure 10:**
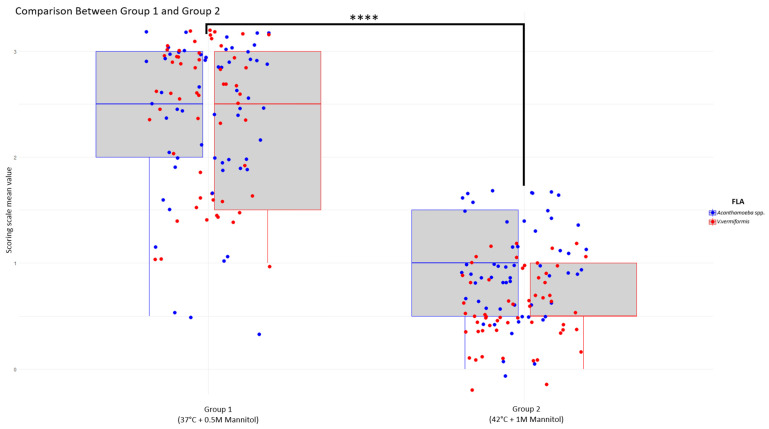
Boxplot, generated in R using ggplot2 (R 4.5.1 version), compares the mean values between group 1 and 2, (*Acanthamoeba* in blue and *Vermamoeba* in red). A noticeable decrease in mean values is observed from Group 1 to Group 2 for both amoebae. Statistical analysis using ANOVA revealed a highly significant difference between groups (**** means *p* = 0.00034).

Based on these considerations, we have differentiated two groups of tests: Group 1, which includes the tests at 37 °C and 0.5 M mannitol, and Group 2, which includes 42 °C and 1 M mannitol. Therefore, both *Acanthamoeba* spp. and *Vermamoeba vermiformis* strain types were found to be osmotolerant and thermotolerant at 37 °C and 0.5 M mannitol, respectively (Group 1). Using our scale of 0 to 3, their average scores were 2.53 ± 0.28 and 2.21 ± 0.18 for *Acanthamoeba* and 2.63 ± 0.45 and 2.16 ± 0.30 for *Vermamoeba vermiformis,* respectively. However, most of them were unable to proliferate at 42 °C and 1 M mannitol (Group 2), with scores of 0.74 ± 0.14 and 0.96 ± 0.12 for *Acanthamoeba* and 0.79 ± 0.04 and 0.37 ± 0.03 for *Vermamoeba,* respectively. No significant differences were observed between *Acanthamoeba* spp. and *Vermamoeba vermiformis* strains; however, clear differences are seen between Group 1 (37 °C/0.5 M mannitol), and Group 2 (42 °C/1 M mannitol) with a *p*-value of *p* = 0.00034 [Figure 10].

## 4. Discussion

The present study provides new insights into the presence and distribution of potentially pathogenic FLA in different environmental sources in Tenerife. The high rate of FLA detection by multiplex qPCR, combined with the successful isolation of diverse amoebic genera through culture methods, highlights the widespread occurrence of these protozoa in soils and water intended for public and agricultural use in Tenerife. Notably, the predominance of *Vermamoeba vermiformis* and *Acanthamoeba* spp., including a significant proportion of the pathogenic T4 genotype, underscores the potential public health implications of environmental exposure to these microorganisms. These findings align with previous global reports while also revealing specific local risks that warrant further investigation.

In a compilation of 104 environmental FLA isolation studies presented by Chaúque et al. (2023) [6], they reported a prevalence of FLA of 62.42% (52.08–72.75) in agricultural soils, a global average of 52.51% [44.94–60.08] for playground soils and a global average of 44% for drinking water. The multiplex qPCR assay demonstrates high analytical sensitivity, allowing the detection of FLA’s DNA at levels corresponding to less than a single cell. With this in mind, our results match the first two types of samples (AS and PS). However, the prevalence in the RW evaluated in our study is higher than the average, despite our RW samples being double treated tap water. This discrepancy may be due to the use of different techniques. While the review only mentions the use of morphological identification methods or conventional PCR, we also used a sensitive method as is the qPCR. Although the qPCR results indicated a high presence of FLA in RW, this was not reflected in culture. This discrepancy may be due to the DNA detected by qPCR originally coming from non-viable cells, possibly as a result of the water temperature or the treatments applied to maintain water safety standards in the fountain system, which are mainly designed for other microorganisms, such as bacteria, for future projects, this issue could be mitigated by performing cellular viability assays on the samples, such as propidium monoazide–qPCR (PMA-qPCR). Samples GHD1S, GHD3S, GHD10S, and GHD13S are of particular interest because they were collected from playground soils and exhibited the highest DNA concentrations (Table S2). This finding suggests a potential increased risk for individuals exposed to these environments, particularly children, who frequent playgrounds and may be more susceptible due to developing immune systems and a higher likelihood of skin injuries.

*Acanthamoeba* is the most frequently reported FLA in both environmental and clinical samples around the world [6,7,36]. It is widely recognized as a pathogenic protozoan responsible for severe infections such as *Acanthamoeba* keratitis, particularly in contact lens wearers, and granulomatous amoebic encephalitis (GAE), primarily affecting immunocompromised individuals [8,37]. Genotype T4 is the most prevalent in both environmental and clinical samples, accounting for approximately 80–90% of cases in global studies [6,38], which is in accordance with the results obtained in our evaluated samples. Some samples showed discrepancies in the detection of genotype T4 when analyzed by qPCR or culture based isolation. This may be due to the presence of inhibitors, particularly in soil samples as has been previously described [39]. The detected genotypes (GHD1S and GHDS18S) and T5 (GHD15SB) have also been associated with cases of *Acanthamoeba* keratitis [9].

On the other hand, similar to our culture based isolation, *V. vermiformis* is typically the second most prevalent FLA in environmental samples [6]. However, the results obtained by qPCR demonstrated that *V. vermiformis* was the FLA that was more commonly detected. Although the difference with *Acanthamoeba* detection is not so high (Table 4), the higher qPCR detection rates of *V. vermiformis* may also be influenced by the predominant life stage present in environmental samples [40]. *Acanthamoeba* readily forms highly resistant cysts in response to environmental stressors [40,41] and its cysts are well-known to be difficult to lyse. This fact could decrease efficient DNA extraction, reducing qPCR sensitivity [40]. In contrast, the *V. vermiformis* cyst wall is less resistant compared to the *Acanthamoeba* cyst wall. Moreover, it is frequently found as a trophozoite stage in anthropogenic water systems such as treated water or biofilms, where conditions may favor its active form [37]. Therefore, as *V. vermiformis* trophozoites and cysts are more susceptible to lysis, DNA extraction is typically more efficient, which may contribute to the higher sensitivity of qPCR assays for this species.

FLA are well known to be able to survive in environmental conditions without the need for a host [1]. Nevertheless, if they accidentally enter an organism, the simple fact of wanting to survive produces the host infection and consequent tissue damage. For FLA to be considered potentially pathogenic, it must be capable of growing at a temperature of at least 37 °C and an osmolarity of 0.1 M mannitol, conditions that simulate the stresses they experience when infecting humans. Tests at 42 °C and 1 M simulate extreme situations, such as fever (a common human defense mechanism against disease) and the high osmotic pressure found in certain tissues, such as mucous membranes. Our findings suggest that our *Acanthamoeba* spp. and *V. vermiformis* isolated strains are capable of surviving stressful conditions within the human body (Group 1), such as the average human temperature (37 °C) and the high osmolarity of tears (approximately 300 mOsm/L [42]). It is important to note that the ability to survive stress conditions does not in itself confirm pathogenicity but rather indicates a potential for pathogenic capabilities. While there are no significant differences between *Acanthamoeba* and *V. vermiformis* strains, Figure 10 indicates that some of the *Acanthamoeba* examined in this study exhibit slightly greater resistance to the adverse conditions present in both groups. Taking into account that these two FLA groups are reported as amoebic keratitis causal agents, this fact represents a remarkable risk. Further investigations, including assays assessing cell or tissue damage, and host–parasite interactions, are required to substantiate their pathogenic potential.

Within the Vahlkampfiidae family, we identified amoebae belonging to three genera: *Vahlkampfia*, *Naegleria*, and *Tetramitus*. No positive samples for *Naegleria fowleri*, the only species in this family currently recognized as a primary pathogen, were detected. To date, its presence has not been reported in environmental samples in Spain. The other Vahlkampfiidae species have been found as co-infections alongside *Acanthamoeba* [43] or as carriers of bacteria responsible for foodborne illnesses [44]. The genus *Rhogostoma* belongs to the phylum Cercozoa, within the Rhizaria subgroup [45]. To date, no FLA from this genus has been reported as pathogenic. However, some studies support its capacity to act as a carrier of bacteria such as *Legionella* [46]. While amoebae belonging to the *Vannella* genus (Previously included in the genus *Platyamoeba* [47]) have been reported as pathogens in fish [48], no studies have confirmed their ability to infect humans. As with the previously mentioned FLA, *Vannella* is also capable of carrying pathogenic bacteria [49]. The locomotive form exhibits one or more pseudopodia with hyaline caps. The floating form displays similar pseudopodia, but they are thinner, longer and more numerous, extending in multiple directions [Figure 7] [22].

## 5. Conclusions

The use of complementary techniques such as qPCR and culture-based isolation allows for a comprehensive view of the presence of FLA in different types of environmental samples. The high prevalence of FLA detected by both qPCR and culture, along with the ability of *Acanthamoeba* spp. and *V. vermiformis* strains to grow at 37 °C and in 0.5 M mannitol, demonstrates that there is a potential risk associated with these environments. Therefore, there are various sectors of the population at risk. Firstly, workers who come into contact with agricultural soil, such as farmers, can become infected with any of the four pathogenic amoebae mentioned previously through skin wounds on their arms and legs, or through accidental contact with their eyes or nasal passages [50]. These infections can lead to diseases such as skin lesions, keratitis or primary amoebic meningoencephalitis (PAM) [1,5,9,16]. Secondly, individuals who consume unprocessed agricultural products or products with poor hygiene standards, as well as water from refrigerated sources, are susceptible to ingesting amoebae harboring bacteria that are resistant to amoebic phagocytosis and capable of causing foodborne illnesses such as salmonellosis, legionellosis, or cholera. Finally, children with underdeveloped immune systems are exposed to similar risks to farmers when playing in playground soils. This risk should be considered not only due to their pathogenic potential but also due to their role as carriers of foodborne disease-causing bacteria. Furthermore, this is the first report of *Balamuthia mandrillaris* in environmental samples from the Canary Islands, Spain.

## Figures and Tables

**Table 1:** Primers used on standard PCR in the present study.

FLA	Forward	Reverse
*V. vermiformis* HV1227F/VERM-RV [23,24]	5′-TTACGAGGTCAGGACACTGT-3′	5′-TGCCTCAAACTTCCATTCGC-3
*Acanthamoeba* spp. JDP1/JDP2 [25]	5′-GGCCCAGATCGTTTACCGTGAA-3′	5′-TCTCACAAGCTGCTAGGGGAGTCA-3′
Vahlkampfiidae VAHL1/VAHL2 [26]	5′-CTCTTCGTAGGTGAACCTGC-3′	5′-CCGCTTACTGATATGCTTAA-3
Universal for FLA FLA-F/FLA-R [27]	5′-CGCGGTAATTCCAGCTCCAATAGC-3′	5′-CAGGTTAAGGTCTCGTTCGTTAAC-3′

**Table 2:** PCR conditions used on standard PCR used in the present study.

Primers	PCR Conditions				
	Initial Denaturation	Denaturation	Annealing	Extension	Final Elongation
*V. vermiformis* Hv1227F [24]/VermR [23]	95 °C—3′	95 °C—20″	58 °C—30″	72 °C—40″	72 °C—10′
		40 Cycles			
*Acanthamoeba* JDP1/JDP2 [25]	95 °C—5′	95 °C—30″	50 °C—30″	72 °C—30″	72 °C—7′
		35 Cycles			
Vahlkampfiidae VAHL1/VAHL2 [26]	95 °C—2′	95 °C—1′	55 °C—1′30″	72 °C—2′	72 °C—7′
		35 Cycles			
Universal for FLA FLAf/FLAr [27]	95 °C—2′	95 °C—30″	58 °C—30″	72 °C—30″	72 °C—7′
		40 cycles			

**Table 3:** Tolerance scoring system.

Score	Tolerance Levels
0	All amoebae are in the cyst stage, and none are outside the initial inoculation zone.
1	Some amoebae are in trophozoite stage and there are amoebae in any stage surrounding the initial inoculation area.
2	A considerable increase in the number of trophozoites, with slight movement across the agar plate.
3	A massive increase in the number of amoebae and widespread extension across the agar plate.

**Table 4:** The qPCR and culture based isolation results.

Sample Type		AS	PS	RW	Total
Total samples (N)		25	20	17	62
qPCR	*Acanthamoeba* (n/%)	14/56%	12/60%	8/47.1%	34/54.8%
	*V. vermiformis* (n/%)	12/48%	17/85%	8/47.1%	37/59.7%
	*B. mandrillaris* (n/%)	2/8%	4/20%	0	6/9.7%
CT Mean value	*Acanthamoeba*	33.32 (IQR: 2.3 Ct)	32.02 (IQR: 1.7 Ct)	33.36 (IQR: 2.9 Ct)	32.74 (IQR: 3.1 Ct)
	*V. vermiformis*	33.52 (IQR: 2.5 Ct)	30.88 (IQR: 2.2 Ct)	33.69 (IQR: 3.4 Ct)	32.43 (IQR: 3.9 Ct)
	*B. mandrillaris*	33 (IQR: 4 Ct)	33.43 (IQR: 3.8 Ct)	-	33.21 (IQR: 2.9 Ct)
Isolation/Culture	Monoxenic cultures	27	17	7	51
	*Acanthamoeba* spp. (n/%)	11/40.7%	4/23.5%	3/42.9%	18/35.3%
	*V. vermiformis* (n/%)	8/29.6%	7/41.2%	2/28.6%	17/33.3%
	Vahlkampfiidae (n/%)	7/25.9%	2/11.8%	2/28.6%	11/21.6%
	Vannellidae (n/%)	1/3.7%	3/17.6%	0	4/7.8%
	*Rhogostoma* sp. (n/%)	0	1/5.9%	0	1/2%

## Data Availability

The original contributions presented in this study are included in the article/Supplementary Materials. Further inquiries can be directed to the corresponding authors.

## Supplementary Materials

The following supporting information can be downloaded at: https://www.mdpi.com/article/10.3390/pathogens15010073/s1, Table S1: Multiplex qPCR primers and probes; Table S2: Complete details for multiplex qPCR results; Table S3: Complete details for culture based isolation results.

## Abbreviations

The following abbreviations are used in this manuscript:

FLA	Free living amoebae
qPCR	Quantitative Polymerase Chain Reaction
NNA	Non nutrient agar
AS	Agricultural Soil
PS	Playground Soil
RW	Refrigerated Water
PAS	Page’s Amoeba Solution or Page’s Saline
