# Supplementary material for: Molecular and Culture-Based Surveillance of Free-Living Amoebae in Human Related Sources in an Outermost Region
Source: Pathogens. 2026 Jan 9;15(1):73. doi: 10.3390/pathogens15010073 (PMC12844781; doi:10.3390/pathogens15010073)
Supplement: Supplementary file 1 [file pathogens-15-00073-s001.zip › pathogens-4068019-supplementary.pdf]

Supplementary Materials:

Table S1. Multiplex qPCR primers and probes.

FLA Species	Primer (forward)	Primer (Reverse)	Probe
***Acanthamoeba* spp. [51]**	AcantF900 (5′-CCC AGA TCG TTT ACC GTG AA-3′)	AcantR1100 (5′-TAA ATA TTA ATG CCC CCA ACT ATC C-3′)	AcantProb (5′-JUN-CT GCC ACC GAA TAC ATT AGC ATG G-QSY-3′)
Vermamoeba vermiformis	Hv1227F (5′-TTA CGA GGT CAG GAC ACT GT- 3′) [24]	VermRv (5′ TGCCTCAAACTTCCATTCGC-3′) [23]	VermProb (5′-ABI-TTG ATT CAG TGG GTG GTG GT-QSY-3′)
***Naegleria fowleri* [51]**	NaeglF192 (3′-GTG CTG AAA CCT AGC TAT TGT AAC TCA GT-5′)	NaeglR344 (5′-CAC TAG AAA AAG CAA ACC TGA AAG G-3′)	BalaProb (5′-6FAM-AG TAC TTC TAC CAA TCC AAC CGC)
***Balamuthia mandrillaris* [51]**	BalaF1451 (5′-TAA CCT GCT AAA TAG TCA TGC CAA T-3′)	BalaR1621 (5′-CAA ACT TCC CTC GGC TAA TCA-3′)	NeglProb (5′-VIC-AT AGC AAT ATA TTC AGG GGA GCT GGG)

Table S2. Complete details for multiplex qPCR results.

Sample Name	Sample type*	Ac	Ac T4	Bm	Nf	Vv
MPP1S	Agricultural soil	32	+	-	-	35
MPP2S	Agricultural soil	34	+	-	-	32,5
MPP3S	Agricultural soil	35	-	-	-	35
MPP4S	Agricultural soil	30	+	33	-	32
MPP5S	Agricultural soil	-	-	-	-	36
MPP6S	Agricultural soil	36	-	-	-	-
MPP7S	Agricultural soil	-	-	-	-	-
MPP8S	Agricultural soil	-	-	-	-	-
MPP9S	Agricultural soil	-	-	-	-	-
MPP10S	Agricultural soil	31,5	+	-	-	31
MPP11S	Agricultural soil	34	-	37	-	33,5
MPP12S	Agricultural soil	36	-	-	-	37
MPP13S	Agricultural soil	33,5	+	-	-	35
MPP14S	Agricultural soil	35	-	-	-	36
MPP15S	Agricultural soil	35,2	+	-	-	36
MPP16S	Agricultural soil	32,2	+	-	-	33
MPP17S	Agricultural soil	34,2	-	-	-	34
MPP18S	Agricultural soil	-	-	-	-	36

MPP19S	Agricultural soil	-	-	-	-	-
MPP20S	Agricultural soil	33,4	+	-	-	32,2
MPP21S	Agricultural soil	37,4	-	-	-	36
MPP22S	Agricultural soil	36	-	33	-	35
MPP23S	Agricultural soil	32,9	-	-	-	36
MPP24S	Agricultural soil	33,6	-	-	-	34
MPP25S	Agricultural soil	-	-	-	-	-
GHD1S	Playground soil	34,5	-	36	-	29,5
GHD2S	Playground soil	32	-	32	-	31
GHD3S	Playground soil	29	-	-	-	28,7
GHD4S	Playground soil	32,5	-	-	-	31
GHD5S	Playground soil	-	-	-	-	31,3
GHD6S	Playground soil	32,3	-	-	-	31,5
GHD7S	Playground soil	35,3	-	-	-	31,7
GHD8S	Playground soil	32,7	-	-	-	31
GHD9S	Playground soil	-	-	-	-	32,5
GHD10S	Playground soil	30,8	-	-	-	29,4
GHD11S	Playground soil	-	-	-	-	-
GHD12S	Playground soil	-	-	-	-	32,8
GHD13S	Playground soil	31	-	32,9	-	28
GHD14S	Playground soil	-	-	-	-	30
GHD15S	Playground soil	-	-	-	-	32
GHD16S	Playground soil	31,8	-	-	-	-
GHD17S	Playground soil		-	-	-	32,9
GHD18S	Playground soil	30,7	-	-	-	31,2
GHD19S	Playground soil		-	33	-	-
GHD20S	Playground soil	31,6	-	35,8	-	30,4
CSR1w	Refrigerated drinking water	-	-	-	-	35
CSR2w	Refrigerated drinking water	-	-	-	-	-
CSR3W	Refrigerated drinking water	-	-	-	-	-
CSR4W	Refrigerated drinking water	34,5	+	-	-	-
CSR5W	Refrigerated drinking water	35	+	-	-	36
CSR6W	Refrigerated drinking water	28,6	+	-	-	29,6
CSR7W	Refrigerated drinking water	30,4	+	-	-	27,8
CSR8W	Refrigerated drinking water	32,7	+	-	-	37,1
CSR9w	Refrigerated drinking water	32,1	+	-	-	34
CSR10W	Refrigerated drinking water	34	+	-	-	35
CSR11W	Refrigerated drinking water	35,7	+	-	-	35
CSR12W	Refrigerated drinking water	37,2	+	-	-	35
CSR13W	Refrigerated drinking water	-	-	-	-	-
CSR14W	Refrigerated drinking water	-	-	-	-	31,6
CSR15W	Refrigerated drinking water	-	-	-	-	-
CSR16w	Refrigerated drinking water	-	-	-	-	-
CSR17W	Refrigerated drinking water	-	-	-	-	-

*Ac: *Acanthamoeba* spp.; Bm: *Balamuthia mandrillaris*; Nf: *Naegleria fowleri*; Vv: *Vermamoeba vermiformis*.

Table S3. Complete details for culture based isolation results.

Isolate	Sample Type	Species	Genotype	Genbank ID
MPP1S	Agricultural soil	*A. castellanii*	T4	PV567158
MPP2S	Agricultural soil	*V. vermiformis*		PP774375
MPP3S	Agricultural soil	*N. americana*		PV567189
MPP4S	Agricultural soil	*Acanthamoeba* sp.	T4	PV567159
MPP5S	Agricultural soil	*T. hohokami*		PV567190
MPP6S	Agricultural soil	*Acanthamoeba* sp.	T4	PV567160
MPP7S	Agricultural soil	*Acanthamoeba* sp.	T4	PV567161
MPP8S	Agricultural soil	*Acanthamoeba* sp.	T11	PV567162
MPP9S	Agricultural soil	*V. vermiformis*		PP774376
MPP10S	Agricultural soil	*V. vermiformis*		PP774374
MPP11S	Agricultural soil	*Acanthamoeba* sp.	T4	PV567163
MPP11SB	Agricultural soil	*T. aberdonicus*		PV567191
MPP12S	Agricultural soil	*V. vermiformis*		PP774377
MPP13S	Agricultural soil	*A. mauritaniensis*	T4	PV567164
MPP14S	Agricultural soil	*Acanthamoeba* sp.	T4	PV567165
MPP15S	Agricultural soil	*V. vermiformis*		PV567166
MPP15S	Agricultural soil	*V. inornata*		PV567192
MPP16S	Agricultural soil	*V. inornata*		PV567193
MPP17S	Agricultural soil	*Acanthamoeba* sp.	T4	PV567167
MPP18S	Agricultural soil	*Acanthamoeba* sp.	T4	PV567168
MPP19S	Agricultural soil	*V. vermiformis*		PP774373
MPP20S	Agricultural soil	*N. americana*		PV567194
MPP21S	Agricultural soil	*V. vermiformis*		PP774378
MPP23S	Agricultural soil	*V. orchilla*		PV567195
MPP24S	Agricultural soil	*Acanthamoeba* sp.	T4	PV567169
MPP24SB	Agricultural soil	*V. vermiformis*		PP774372
MPP25S	Agricultural soil	*P. placida*		PV567170
CSR2w	Water	*Acanthamoeba* sp.	T4	
CSR5W	Water	*A. castellanii*	T4	PV567185
CSR6W	Water	*T. aberdonicus*		PV567199
CSR7W	Water	*V. vermiformis*		PV567186
CSR8W	Water	*N. indonesiensis*		PV567200
CSR10W	Water	*V. vermiformis*		PV567187
CSR11W	Water	*A. castellanii*	T4	PV567188
GHD1S	Playground soil	*Acanthamoeba* sp.	T2/6B	PV567171
GHD1SB	Playground soil	*V. vermiformis*		PV567172
GHD2S	Playground soil	*P. placida*		PV567173
GHD3S	Playground soil	*V. vermiformis*		PV567174
GHD4S	Playground soil	*R. florae*		PV567196
GHD5S	Playground soil	*V. vermiformis*		PV567175
GHD6S	Playground soil	*P. placida*		PV567176

GHD7S	Playground soil	*A. castellanii*	T4	PV567177
GHD9S	Playground soil	*V. vermiformis*		PV567178
GHD11S	Playground soil	*T. rhodos*		PV567197
GHD12S	Playground soil	*V. vermiformis*		PV567179
GHD14S	Playground soil	*V. vermiformis*		PV567180
GHD15S	Playground soil	*T. rhodos*		PV567198
GHD15SB	Playground soil	*A. lenticulata*	T5	PV567181
GHD16S	Playground soil	*P. placida*		PV567182
GHD18S	Playground soil	*Acanthamoeba* sp.	T2/6B	PV567183
GHD20S	Playground soil	*V. vermiformis*		PV567184
